# Looking at Developmental Neurotoxicity Testing from the Perspective of an Invertebrate Embryo

**DOI:** 10.3390/ijms23031871

**Published:** 2022-02-07

**Authors:** Gerd Bicker

**Affiliations:** Institute of Physiology and Cell Biology, University of Veterinary Medicine Hannover, Bischofsholer Damm 15/102, 30173 Hannover, Germany; gerd.bicker.iR@tiho-hannover.de

**Keywords:** axonal pathfinding, embryo culture, semaphorin, directed cell migration, *Locusta migratoria*

## Abstract

Developmental neurotoxicity (DNT) of chemical compounds disrupts the formation of a normal brain. There is impressive progress in the development of alternative testing methods for DNT potential in chemicals, some of which also incorporate invertebrate animals. This review briefly touches upon studies on the genetically tractable model organisms of *Caenorhabditis elegans* and *Drosophila melanogaster* about the action of specific developmental neurotoxicants. The formation of a functional nervous system requires precisely timed axonal pathfinding to the correct cellular targets. To address this complex key event, our lab developed an alternative assay using a serum-free culture of intact locust embryos. The first neural pathways in the leg of embryonic locusts are established by a pair of afferent pioneer neurons which use guidance cues from membrane-bound and diffusible semaphorin proteins. In a systematic approach according to recommendations for alternative testing, the embryo assay quantifies defects in pioneer navigation after exposure to a panel of recognized test compounds for DNT. The outcome indicates a high predictability for test-compound classification. Since the pyramidal neurons of the mammalian cortex also use a semaphorin gradient for neurite guidance, the assay is based on evolutionary conserved cellular mechanisms, supporting its relevance for cortical development.

## 1. Introduction

Growing concerns about chemical compounds that potentially cause an increase in neurodevelopmental disorders has stimulated research in developmental neurotoxicity. This is a rather challenging field within applied and basic science, requiring epidemiological studies, neuropsychological investigations, biomedical brain imaging, and toxicological data, including modern omics-based and in silico approaches about the effects of neurotoxicants on the developing nervous system. While neurotoxicity has been defined as “any adverse effect on the chemistry, structure or function of the nervous system during development or at maturity, induced by chemical or physical influences” [1], developmental neurotoxicity (DNT) especially addresses the problem that, during its growth, the human brain is more sensitive to chemical exposure than during adulthood [2,3,4]. Thus, a large part of DNT research is focusing on methods to predict the impact of chemicals on the embryo or fetus. Apart from ethical considerations within the framework of the 3Rs Principle [5], current recommendations for DNT testing using rodents [6], are time consuming and require large numbers of test animals. The last decade has brought tremendous progress in the scientific effort to advance new, alternative methods for DNT testing [7,8,9,10,11,12,13,14], yet obstacles remain for the incorporation of these methods into mandatory regulatory safety assessment. Additional efforts are dedicated to the inclusion of the adverse outcome pathways (AOP) concept in DNT testing, in which a molecular initiating event subsequently leads to impairments in a chain of key developmental processes, eventually resulting in an adverse outcome [15]. A recent review has outlined the improvements that international stakeholders have made in the development of a test battery comprising various human and rat cell-derived in vitro models [16]. This in vitro battery has been devised for the analysis of key neurodevelopmental events, including neuroprogenitor proliferation, cell migration, and differentiation into neuronal and glial cell types, neurite outgrowth, synaptogenesis, and formation of an electrically active network. It will improve DNT testing by taking adverse outcome pathways (AOPs) into account. Moreover, the assays of the stakeholders have been evaluated for readiness of use [16].

DNT testing aims to protect the developing human nervous system. For natural reasons, practitioners in the field of neurotoxicology have culture models of human, mammalian, or vertebrate cells foremost in their mind. Some researchers also considered the inclusion of certain invertebrate animals into toxicological testing [17,18,19,20]. Invertebrates have a long and impressive history in biomedical research. A comprehensive review has covered the scientific history and the main contributions to biomedical progress of the many diverse organisms, as evidenced by an impressive number of Nobel Prizes for research on invertebrates [21]. There are immediately plausible reasons for using invertebrates, such as the rapid read-out of behavioral disorders after exposure of intact animals to toxicants, enabling for high throughput screens. The mechanistic underpinnings of nervous system development are best understood in two species. These are the fly *Drosophila melanogaster* and *Caenorhabditis elegans*, a soil nematode worm. *C. elegans* was the first animal with a completely sequenced genome, achieved in the year 1998, followed by *Drosophila* in 2000 [22,23]. Apart from their well-annotated genomes, their main advantage as lab animals is short generation time, easy screening for mutants, a versatile toolbox for cell-lineage tracing and genetic manipulation, availability of connectivity diagrams for the nervous system, and the presence of sets of identifiable neurons [24,25,26,27,28]. Essential arguments for using an invertebrate model system in toxicological studies, instead of a mammal [19,29], are evolutionary conserved molecular mechanisms for development and cellular functions. Due to the phylogenetic distance, ethical concerns are also reduced.

Despite the fact that a considerable part of the invertebrate community is involved in research about animal models for human diseases [24,30,31,32,33], the advantage of using available model organisms for DNT testing has hardly been recognized. Except for many applied studies of ecotoxicological risk assessment [34,35,36], which are not in the focus of my review, the issue of DNT did not catch the attention of most invertebrate developmental neurobiologists. This is quite understandable because, even in toxicology, DNT is among the most difficult and least-studied areas [10]. My aim is to encourage developmental biologists to cross a divide between existing fields and foster interdisciplinary research with toxicologists. I propose that certain invertebrates might provide suitable models for DNT risk assessment. Especially assays using intact animals should be considered as alternative models in DNT test batteries. 

A disadvantage of cell culture research is the isolated cellular environment, remote from interactions with the whole organism. This approach allows only for the study of cell-intrinsic developmental mechanisms. An important feature of development in metazoan organisms is, however, the interaction of different cell types. Using intact invertebrates in DNT studies can nicely combine the accessibility for test substances of in vitro studies with the advantage for providing an in vivo organismal development. My review briefly covers selected examples of DNT studies in worm and fly. Further to this, main parts describe in more details a recent approach to design a DNT-assay for correct anatomical wiring, using another classical model system of developmental neurobiology. This approach follows the recommendations for the selection of reference compounds and appropriate use in alternative DNT assays [7]. Intact locust embryos are cultured in a serum-free medium to analyze DNT effects of chemical compounds on axonal pathfinding of a pair of identified neurons [37,38]. Results from a panel of established DNT-positive and DNT-negative test compounds support a rather high level of human predictability for this assay.

## 2. DNT Studies in Two Genetic Model Systems

### 2.1. DNT Studies in C. elegans

*C. elegans* is one of the preferred invertebrate test organisms for toxicology. Its biggest advantages for DNT studies are the small number of 302 neurons and 56 glial cells (for the hermaphrodite worm), a high degree of genetic homology between the nematode and the human genome, and a complete connectome [20,28,29]. For behavioral analysis in oral toxicity evaluations, a so-called Development and Activity Test has been designed [39]. It is based on recording motor activity in neurotoxicant-exposed populations. During worm development, the progression through each of the four larval stages requires the shedding of the cuticle while locomotion and food uptake stop. The periodic levels of motor activity and inactivity of exposed worms are recorded and compared to those of controls. Developmental effects are reflected in time-shifted activity rhythms and changes in peak amplitudes as indicators of stage-specific motor behavior. The heavy metals, arsenic, lead, and mercury, led to developmental delay and hyperactivity, while lithium reduced activity at concentrations that caused little developmental delay [39]. Correspondingly, the heavy metals are already known as established developmental neurotoxicants associated with hyperactivity in children, while lithium is not. An impressive sample of developmental neurotoxicants, including heavy metals, fluoride, and organophosphorus pesticides, have been examined for developmentally delayed effects on behavior and neurodegeneration of specific neurons with genetically labeled transmitter phenotypes [20]. Developmental exposure to an increasing concentration of nickel chloride caused degeneration of cholinergic, dopaminergic, and GABAergic neurons [40]. Changes in functional motor response and basal slowing response reflected the degeneration of cholinergic and dopaminergic neurons. A characteristic behavioral phenotype in a small number of nickel-exposed worms can be interpreted as a reduced function of a specific subset of GABAergic neurons. Expression of GFP-tagged glutathione S-transferase 4 indicated that neural damage by nickel exposure was due to oxidative stress [40].

### 2.2. DNT Studies in Drosophila

The need for alternative models in toxicological studies has also advanced research using *Drosophila*. After embryogenesis, *Drosophila* passes through three larval instars before pupating, undergoing metamorphosis, and eclosing as an adult insect. A review has outlined experimental methods and sensitive endpoints of “Drosophotoxicology” [18]. Toxicant exposure is possible for the fly embryo and developing larvae. Dose-dependent larval exposure to methyl mercury, mercury chloride, lead acetate, and sodium arsenite induced larval lethality and reduced the time-delayed pupariation, metamorphosis, and eclosion, with methyl mercury as the most toxic metal compound [41]. This model also proved insightful for the study of metal mixtures. It is difficult to dissect the mechanisms of methyl-mercury-induced developmental toxicity due to a wide variety of adverse effects in humans. Larval exposure in *Drosophila* resulted in morphological muscle defects which correlated with failed adult eclosion [42]. Methyl mercury exposure caused enhanced expression of an antioxidant reporter construct in the muscles. To characterize antioxidant signaling by the *Drosophila* orthologue of Nrf2 transcription factor as protective measure, adult eclosion behavior was recorded in parallel with morphology of two muscle groups. Muscle- and neuron-specific genetic manipulation showed tissue-specific, but independent protective functions of the Nrf2 signaling pathway against developmental toxicity [42].

There are a number of studies dedicated to the field of alcohol-induced developmental neurotoxicity. Pregnant mothers should avoid consuming alcohol because it causes adverse effects in the fetus, can easily cross the blood–brain barrier, and leads to deficient growth of the developing brain [43]. To mimic aspects of the fetal alcohol syndrome in *Drosophila,* both larval and embryonic exposure can serve as models [44,45].The most common method is voluntary alcohol uptake via food during the larval stages, during which the whole organism grows enormously. *Drosophila* reared on ethanol-containing food showed a dose-dependent developmental delay; reduced size of larval CNS, eclosion, and size as adults; and altered behavioral responses to alcohol vapor [44]. Developmental exposure to ethanol causes many types of adverse effects in mammals, including the reduced expression of insulin, insulin-like growth factor II, and insulin-like growth factor receptors in the brain [46]. Similar to that of mammals, organ growth in *Drosophila* is regulated by insulin-signaling pathways, and ethanol-induced phenotypes are due to reduced expression of insulin-like peptides [44]. Transgenic expression of insulin-like peptides in the larval brain rescued both the ethanol-induced developmental and behavioral defects in adults.

Since the embryo is protected by a chorion layer and vitelline membrane, a chemical dechorionation step is required before alcohol exposure during embryogenesis. Internal ethanol concentration can be determined by homogenization of the embryos and measurements of supernatant in an alcohol analyzer [45]. Dose-response curves showed a decrease in adult viability after embryonic exposure. This application method revealed specific reduced motor axon outgrowth in ethanol-treated embryos. The treatment caused embryonic pathfinding errors due to an abnormal fasciculation pattern of the identified motor nerves at a concentration when adult viability was still high. Moreover, the density of sensory neuron clusters was also affected. The defects in embryonic neurodevelopment became manifest as changes in larval behavior, resembling disruptions in the developing sensory cortex found in mammalian fetal alcohol syndrome studies [45,47]. Methanol exposure is highly toxic to the *Drosophila* embryo [48]. At a concentration (4%) where lethality increased to 30-40%, about half of those embryos expressed severe anatomical defects in the CNS. These included disruptions of the axonal scaffold and aberrant neuronal location. Cell divisions of neuronal precursors were not affected, but cell movements and apoptosis are likely to contribute to the defects. The involvement of apoptosis is supported by an analysis of mutants lacking cell death genes, which show milder defects after methanol treatment [48].

In summary, invertebrate genetic model systems are very convenient for elucidating the mechanistic bases of developmental toxicity and DNT. To advance the models, multiple chemicals, including established DNT-negative compounds, should be tested. This would allow an estimate of their predictability for DNT classification.

## 3. Axonal Pathfinding in Locust Embryo

**Figure 1:**
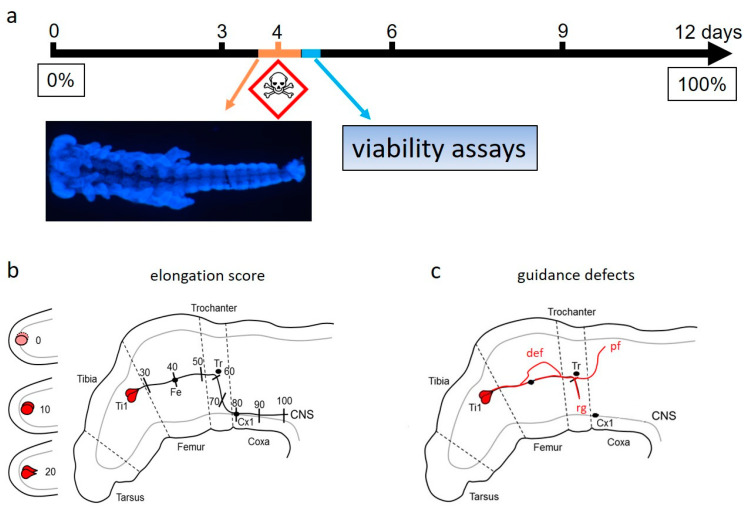
DNT assay system for axonal elongation and guidance defects of Ti1 pioneer neurons in embryos of *Locusta migratoria*. The figure is taken with permission from [38]: (**a**). Timeline of embryonic development from fertilization (day 0, 0%) to hatching (day 12, 100%) at a temperature of 30 °C. Cultured embryos were incubated in test compounds at the end of day 3 for 18 h (orange tag), followed by two different biochemical assays (blue tag) for viability. Fluorescence image shows a DAPI stained embryo with anterior to the left. (**b**)**.** Elongation score for quantifying the length of pioneer axons in the limb bud is shown in a scoring scheme ranging from 0 to 100 [37]. Soma positions of the two Ti1 pioneer neuron in the tibia, femur Fe guidepost cell, trochanter Tr guidepost cell and the two coxa Cx1 guidepost cells serve as reference points. (**c**). Chemical-induced guidance defects are scored, including defasciculations (def) of the two axons, pathfinding errors (pf), and retarded growth (rg).

One of the fundamental issues of brain development is the specificity of the anatomical wiring. In other words, how do neurons find their correct target cells, or, who talks to whom? Neurons elongate their nascent axons by a growth cone positioned at the axon tip. The growth cone is a motile structure that senses chemical cues of the tissue environment. Multiple cues are integrated by the signal transduction pathways of the growth cone, which change cytoskeleton organization, ultimately resulting in motility changes and goal-directed navigation. To devise an assay for the detection of developmental neurotoxicants targeting correct axonal outgrowth, my lab recently turned to embryonic locusts (Figure 1), which have fascinated developmental neurobiologists for many years. The relatively transparent nervous system of the locust embryo is an attractive preparation for investigating cell lineage, the generation of uniquely identifiable neurons, growth cone guidance, and transmitter choice during development [49,50,51,52]. Due to whole embryo culture, live optical imaging, and the use of cell-specific markers, several mechanistic principles of axon guidance have been elucidated which are shared by developing vertebrate nervous systems.

Pioneer neurons establish the first axonal pathways that are followed by later-growing axons. This cell type has been found both in invertebrate and vertebrate nervous systems [53,54,55]. In the limb buds of locust embryos, identified pairs of peripheral pioneer neurons, termed the tibial (Ti1) pioneers, elongate their axons along a precisely defined pathway from their origin near the tip of each appendage to the CNS [53,54] (Figure 1). The axons of later-differentiating limb sensory neurons follow and fasciculate with the pioneer axons. If the pioneer neurons are prevented from differentiating by heat shock, then the sensory neurons fail to reach the central nervous system [56]. The Ti1 pioneers are therefore essential for the guidance of later-differentiating limb neurons.

We considered these pioneer neurons for DNT studies because, in this simple experimental system, some of the complex cellular strategies for growth cone guidance have been carefully worked out. At about 30% of embryonic development [57], the sibling pioneer neurons arise by division of a single mother cell in the epithelium at the tip of the limb [54,58]. The two pioneer axons use the epithelium and basal lamina as substrate for their outgrowth [59]. During outgrowth, the sibling neurons remain in close contact, and the axons fasciculate. The pioneer neurons express the cell-surface glycoprotein Fasciclin I, which contains an evolutionary-conserved domain, common to a vertebrate and invertebrate protein family [60]. Denaturation of Fasciclin I by chromophore-assisted laser inactivation disrupts axonal adhesion for the Ti1 pioneers, suggesting that this protein functions as a cell-adhesion protein [61]. Fasciclin II is another glycoprotein protein expressed by the developing pioneer neurons. It is a member of the immunoglobulin superfamily, with a structural similarity to the neural cell adhesion molecule (N-CAM). A refined method with more focused laser pulses and specific timing of chromophore-assisted laser inactivation revealed that Fasciclin II is required for the initiation of Ti1 axon outgrowth with no effect on axon adhesion. Inactivation of Fasciclin I, in turn, prevents axon adhesion, but not axon initiation [62].

On their track towards the CNS, the pioneer growth cones encounter intermediate targets, the so-called guidepost cells. These guidepost cells are nascent afferent neurons whose axons will later travel to the central nervous system by fasciculating with the pioneers [54]. Two guidepost cells especially contribute to pathfinding towards the CNS [63]. A characteristic feature of the pioneer pathway is the abrupt ventral turn of the axons that are delineated by the trochanter and two coxa guidepost cells (Figure 1b).

The position of this ventrally directed axon also coincides with the epithelial expression of a semaphorin protein [64]. Semaphorins belong to a family of proteins implicated in axonal guidance, and which contain the conserved Sema domain [65]. The first characterization of a semaphorin (Sema I, a transmembrane protein originally termed ‘Fasciclin IV’) showed expression in epithelial stripes of the locust limb bud, including the circumferential band at the trochanter segment [64]. Antibody-blocking experiments revealed disruption of the characteristic ventral turn of the pioneer axons [64]. A gain-of-function approach showed that ectopic expression of the extracellular domain of recombinant semaphorin I acted as an attractive guidance cue for the pioneers [66]. Other identified guidance cues are the dorso-ventrally and distal-proximally oriented gradients of the secreted semaphorin molecule Sema-2a. These gradients repel the pioneer growth cones away from the periphery and contribute to the guidance towards the CNS [67].

The seminal discoveries of membrane-bound and diffusible semaphorin cell recognition molecules expressed in embryonic locusts [64,67] prompted our lab to explore this preparation as a DNT testing system for the accuracy of axonal outgrowth [37]. In the mammalian cerebral cortex, another member of the semaphorin family (Sema 3A) is distributed as a molecular gradient to instruct the oppositely directed outgrowth of dendrites and axons of pyramidal neurons [68]. Pyramidal neurons provide the excitatory output of the cortex, comprising about 70–85% of its total neurons [69]. Since many molecular guidance cues, cytoskeletal molecules, and signal transduction pathways are evolutionary conserved [64,65,67,68], we suspected that disrupting the pioneer axon pathway by chemicals might also reveal information about their DNT potential on mammalian brain circuitry formation.

## 4. DNT Test Assay

During the lifecycle, locust females lay their eggs as egg pods into the soil. The egg pods are held together and protected by a foam-like secretion. The embryo feeds on the egg yolk, then hatches as a worm-shaped larva and develops over five freely moving grasshopper stages into an adult [49]. Locust embryos can be relatively easily dissected out of the egg and kept intact in tissue culture. As another experimental advantage, the approximately 50–60 eggs of one pod can be split into different experimental and control groups of the same age. Embryos in a single clutch usually differ by only 1% of development from each other. To devise an animal-free test system, we cultured the embryo in a serum-free Leibovitz L-15 medium, which is an advancement over the classical culture method using serum-supplemented RPMI [70]. This whole-embryo culture system was used because a culture of dissociated locust neurons in serum-free L-15 also supported neurite outgrowth [71,72]. At a stage of about 32.5 % of development [57], locust embryos of one egg pod were subdivided into three different groups. These are the start groups, medium control groups, and test compound groups. Start groups were immediately fixed and used to determine the length of axons in the beginning of the experiment and for normalization of concentration–response curves among experiments. Medium control groups were cultured in serum-free Leibovitz L15 for 18 h without containing test chemicals. The test compound groups were incubated with established DNT-positive or DNT-negative compounds. To determine acute toxicity on the embryo, we determined viability of the locust embryo with resazurin and dead cell protease assays for necrosis. Using immunofluorescence microscopy methods with an antibody specific for insect nervous systems [73], we visualized the pioneer neurons of the cultured embryos. Pioneer axon extension was quantified as an elongation score using landmarks, such as the guidepost cells or leg-segment borders [37]. For each hind leg, the endpoint of axon extension was scored between 0 and 100% (Figure 1b).

Guidance defects were evaluated by the following criteria (Figure 1c). The separation of both axons over a distance of 10% was counted as a defasciculation error (def error, Figure 1c). Leaving the stereotypical pathway by one or both axons was counted as an error in pathfinding (pf error, Figure 1c). Axons that did not elongate to the average length of the control group were considered as retarded growth (rg error, Figure 1c).

After normalization, the pooled values for the four endpoints, axon elongation, correct pathfinding, metabolic viability, and necrosis, were plotted in concentration–response curves. These curves served to determine IC50 values and most sensitive endpoints according to [11,74]. To assign a compound as a developmental neurotoxicant, the curves for axon elongation and correct pathfinding were compared to those of viability in a resazurin assay and a necrosis assay. A prediction model, details of the statistical methods, and criteria for assigning a distinction between general toxicity versus developmental toxicity were described in [38].

## 5. Results of DNT Assay

According to recommendations [4,9], we selected established DNT-positive and DNT-negative compounds for performance in the pioneer axon assay system [38]. A summary of the results is shown in Table 1.

From a training set of 12 selected compounds, representing heavy metals, pesticides, pharmaceuticals, and general cytototoxins, 10 compounds fulfilled the predicted outcome. Established developmental neurotoxicants, such as methyl mercury, arsenic, and the anti-epileptic agent valproic acid, also proved DNT-positive in the insect assay. In accordance with its selective inhibitory effects on the axonal outgrowth of human neurons [11], the mitochondrial respiratory chain blocker rotenone classified as a specific developmental neurotoxicant for pioneer axon elongation and navigation [37,38]. Apart from blocking the respiratory chain complex I, rotenone also interferes with the cytoskeletal dynamics of microtubule assembly and the RhoA/ROCK pathway [75,76]. Small molecule blockers of the Rho/ROCK pathway promote neurite extension of cultured human model neurons [11,77,78]. The ROCK inhibitor Y27632 partially restored the rotenone-induced decrement of pioneer axon elongation [37]. This rescue experiment supports the classification of rotenone as a specific developmental neurotoxicant. Quantitative data about the IC50 of the various concentration response curves are listed in Table 2.

The organophosphate chlorpyrifos is a blocker of acetylcholine esterase enzyme (AChE), which terminates cholinergic neurotransmission by hydrolyzing acetylcholine [2,79]. Somewhat counter-intuitively, the DNT-positive pesticide chlorpyrifos and its liver metabolite chlorpyrifos oxon are negative in the pioneer axon assay [2,38,79,80]. Even though the CNS and peripheral mechanosensory neurons develop high AChE levels during locust embryogenesis, we detected no expression in the pioneer neurons [72]. This finding may explain the incorrect classification in the pioneer neuron assay. In addition, it demonstrates quite clearly that DNT studies on a single neuron are not sufficient and that future investigation must be expanded to identified neurons in other parts of the nervous system. In summary, our current approach identified 83% of the tested compounds correctly. An incorrect classification of chlorpyrifos or chlorpyrifos oxon was also found in other in vitro models, such as a human mesencephalic precursor cell line (LUHMES) for neurite outgrowth or neurosphere assays of rat and human neurons for differentiation, migration, and proliferation [11,74]. It will be essential in future work to expand the number of compounds and to include more insecticides with other modes of action into the assay, such as pyrethroids, acting on the permeability of sodium channels.

Studies in intact animals face the problems of undefined actual concentrations in the tissue of interest due to diffusion barriers and metabolic turnover. In the standard assay, two embryos of a volume of 0.033 µL each [81], are cultured together in a single well containing an incubation volume of 200 µL of test compounds dissolved in medium. A comparison of the rather small tissue volume to the incubation volume suggests that the embryonic tissue is not capable of metabolizing or chelating large proportions of the test compounds. Most likely, the values in the obtained concentration–response curves represent effective concentrations.

## 6. Endpoint-Specific Controls

Establishment of DNT assays require endpoint-specific controls with known mechanistic actions [7]. Growth cone motility driving axonal extension should be affected by blocking cytoskeletal dynamics. We determined the effects of the actin polymerization inhibitor cytochalasin D and the microtubule inhibitor colchicine on axon elongation and found a concentration-dependent reduction while general viability did not decrease [37].

Cytoskeletal dynamics in migrating growth cones is highly dependent on alterations in cytosolic calcium levels, which also applies for pioneer neurons [82,83]. Because the L-type calcium channel inhibitors verapamil and diltiazem are also effective in the insect nervous system [84], we assayed both compounds. Both drugs reduced pioneer axon elongation in a dose-dependent manner, but a comparison with the curves for viability revealed an endpoint-specific DNT-positive classification [37].

## 7. Testing Unclassified Compounds

In a systematic literature review about in vitro and alternative DNT testing methods [85], the pro-apoptotic alkaloid staurosporine was listed as a possible DNT-positive compound. This classification was in line with test results from the pioneer neuron assay, suggesting that staurosporine is indeed DNT-positive [81]. A differential distribution of the cGMP synthesizing enzyme, soluble guanylyl cyclase (sGC), in dendrites and axons of pyramidal neurons effects the opposite response of these neurites to the semaphorin gradient in the mammalian cortex [68]. The assembly of a localized cGMP-synthesis complex by a scaffolding protein is also necessary for apical dendrite development in embryonic pyramidal neurons of the hippocampus [86]. While an in vivo link between semaphorin 1a-mediated motor axon guidance and alteration of intracellular neuronal cGMP levels has been shown for *Drosophila* [87], currently we have no detailed information about the signal transduction pathways downstream of semaphorin reception in the leg pioneer neurons. Using the specific inhibitor of soluble guanylyl cyclase ODQ, we found navigation errors and reduced axon elongation of the pioneer neurons, whereas the viability remained unaffected [38,81]. Because ODQ completely disrupts oriented dendritic outgrowth without affecting dendritogenesis [68], the combined data from mammalian cortex and locust pioneers classify the drug ODQ as DNT-positive. These results are also consistent with the DNT-positive outcome of ODQ application on pioneer axon growth in the locust olfactory system [88], on the cellular migration of developing human model neurons [89], and on fetal human neural progenitors [90]. Krug et al. classified ODQ as unspecific on neurite outgrowth of differentiating human LUHMES neurons in cell culture [11]. The enzyme sGC is the physiological receptor molecule for the messenger NO. To reveal the inhibitory action of the blocker on neurite outgrowth, sGC activity presumably depends on the release of NO, which is most likely absent in the cell culture experiments [11]. Chemoneuroanatomical methods show that NO can be released from surrounding epithelial cells onto cGMP-synthesizing pioneer neurons in the embryonic olfactory system of the locust [88]. Even though neurite outgrowth is rather easy to measure as a functional endpoint in the artificial environment of cell culture, assays performed on tissue of intact embryos appear to be more revealing. Complementary approaches using intact tissue of other insects could reinforce this view. For example, sensory neurons expressing NO-induced cGMP-immunoreactivity have also been discovered on developing imaginal discs in *Drosophila* [91]. Tissue culture methods for intact imaginal discs that support neurite outgrowth are also available [92].

## 8. Limitations of the Pioneer Neuron Assay and Future Research

### 8.1. Limitations and Avenues for Future Improvements

Successful DNT-assessment has to strike a balance between rapidity of performance for testing many chemicals and the accuracy with which the toxicological assays reflect the key developmental processes of the nervous system. The pioneer assay is fast enough to avoid chemical decay, but it allows for the read-out of the essential morphological endpoints in a critical time window. To provide a quantification of axon extension and pathfinding error in concentration–response curves, we relied on a rapid scoring scheme based on landmarks [37]. This method is largely independent of the exact limb bud orientation in the focal plane of the fluorescence microscope and a trade-off between the accurate determination of axon length in 3D images and speed of assay quantification.

Scanning laser optical tomography (SLOT) is a novel and relatively fast 3D-imaging technology which, at first, was applied for the reconstruction of fluorescent neurons in cleared tissue of larval locusts and *Drosophila* [93]. In an improved set-up, the resolution of SLOT was also sufficient to determine the exact anatomical shape of the pioneer neurons in locust embryos [81]. A segmentation algorithm was used to outline the 3D shape of the pioneers. So far, this algorithm has required the manual setting of a fluorescence intensity threshold for the removal of background. A technical advance would be a fully automated image recognition tool for a more rapid and completely unbiased evaluation of the pioneer neuron shape. SLOT imaging of a completely cleared and embedded embryo required about 45 min. A more rapid data acquisition by other optical 3D-imaging methods, such as light sheet microscopy, should also be explored.

Locust embryos were incubated in the standard assay with DNT-positive test compounds to compare the resolution of pioneer neuron anatomy using conventional fluorescence microscopy and SLOT [81]. Both methods applied to identical embryos yielded a similar outcome. The results confirmed again the DNT-positive classification of methyl mercury and arsenite. SLOT also resolved axonal navigation errors and multiple abnormal neurites emanating from the cell bodies of arsenite-exposed pioneer neurons.

One advantage of the pioneer axon assay is the quantification of a stereotyped pathway taken by the axons of single, identified neurons. This may also be regarded as a disadvantage, depending on the view, because the quantification of the general toxicity requires biochemical measurements on complete embryos. It would be informative for a mechanistic analysis to measure directly cytotoxic effects on the pioneer neurons. The introduction of fluorescent assays for cytotoxicity determination in single cells, such as TUNEL staining, live-cell imaging of induced cell death [94], or calcium imaging may help to resolve this issue. The first barrier against a toxic insult is the intact cell membrane. Rapid rises of cytoplasmic calcium levels reflect a leak of the steep calcium gradient across the membrane and are good indicators of membrane breakdown. However, the injection of calcium indicators for tracking the flow of calcium into a single pioneer neuron requires a time-consuming preparation of the embryo as a limb fillet [83]. Recently, we found that the next-generation calcium indicator dye Cal-520 AM could be bulk-loaded into locust neurons [95]. This technique has the potential for application to the intact embryo to visualize calcium signals in the pioneers.

### 8.2. Inclusion of Additional Endpoints and Potential for AOP Development

The directed migration of neural precursor cells from proliferative zones to their final destination is one of the key processes in brain development [7,96]. The majority of alternative DNT-assays using mammalian neurons measured mainly cell motility, but not directed migration. A rather impressive example of cell migration is the colonization of the gastrointestinal tract by neural-crest-derived progenitors that eventually form the enteric nervous system [97]. Analogous processes also apply to the formation of the enteric nervous system in large insects [98,99]. In the locust embryo, the midgut nerve plexus is populated by the directed migration of postmitotic, but immature, enteric neurons [98]. The enteric neurons are born in a neurogenic zone at the foregut–midgut boundary. Subsequently, they migrate posteriorly on four stereotyped pathways and extend terminal synaptic branches into the midgut musculature [98,100]. These developmental events happen after completion of more than half of embryogenesis. To overcome the already-formed water-impermeable cuticle, a small incision directly above the foregut allows for access of pharmacological agents to the gut during embryo culture. Using this preparation for pharmacological manipulation, we could show the functional role of NO/cGMP, cAMP, and CO-signal transduction as regulators of cell migration [101,102]. A positive regulatory role of NO/cGMP signaling, characteristic for directed neuron migration along defined pathways on the gut, but not for convergent cell migration during the formation of enteric ganglia, demonstrates the selectivity of the pharmacological test system [103]. To conclude, the time is ripe to incorporate neuron migration on the midgut as an additional assay for DNT-testing.

Approaches for DNT detection should include several endpoints (Table 3) that simulate key developmental processes [104]. Key processes in the developmental of the locust leg nervous system are neurogenesis and apoptosis of the transient pioneers. These events were characterized at single cell levels and can be detected by immunocytochemical staining for expression of a neuronal antigen and the above-mentioned cytotoxicity assays [54,105]. An overview about the expression of transmitter phenotypes and their receptors during embryonic development of the CNS has already been published [49]. Table 3 compiles current and future research projects about endpoints and AOPs, including references from our and other research groups.

Currently, the read-out of the pioneer neuron assay is confined to the time window of axon outgrowth on the limb bud (Figure 1). Using specific antibodies, it is possible to include axonal outgrowth of later differentiating pioneer neurons in the CNS or peripheral mechanosensory neurons. This would expand the temporal range of testing for the disruption of neural connectivity. Good candidates would be the differentiation of stretch receptors in the wing hinges [106], which express AChE [72] (Table 3). Intracellular recordings from the soma revealed electrical excitability at 60% and overshooting action potentials at 70% of embryogenesis [106]. The forewing mechanoreceptor makes a monosynaptic connection to the first basalar motoneuron. Stimulating the adult stretch receptor axon with a suction electrode in an afferent nerve and intracellular recordings from the motoneuron soma under visual control in an isolated ventral nerve cord preparation uncovered a cholinergic excitatory postsynaptic potential [107]. An adaptation of these techniques to early locust development would allow for DNT testing of synaptogenesis between identified neurons as a realistic goal.

### 8.3. Extrapolation from the Invertebrate Model to Humans

How can results from an insect embryo be translated to quantitative data about risk assessment for the human organism? In modern toxicology, there are specific quantitative in vivo-to-in vitro extrapolations (QIVIVE) in development which combine bioassay data with physiologically based kinetic modelling [108,109]. Based on the consideration of the rather small embryonic and large incubation volume [81], the effective concentrations of our applied test compounds are most likely correct for the insect nervous system. However, in late-stage embryos, the development of a blood–brain barrier (in insect terms called hemolymph–brain barrier) has to be taken into account. There is also the problem of the metabolic breakdown of the applied chemicals by cytochrome P450 enzymes. Due to contributions from other organs, such as the liver, the nervous system intrinsic metabolism of compounds is rather difficult to characterize in most animals. To predict brain uptake of drugs in vertebrates, a novel ex vivo model using the locust brain has been developed [110]. In this experimental approach, the small insect brain with a relatively large surface is placed in a well containing the test compound dissolved in buffer. After standard exposure time, the compound concentration is determined by liquid chromatography–mass spectrometry. Measuring the uptake of 25 known drugs over the hemolymph–brain barrier results in linear correlation with in situ perfusion data from vertebrates. The locust brain model could also recognize P-glycoprotein (PgP) transporter substrates [110]. Kinetic analysis allows for the separate evaluation of barrier permeability, efflux, drug metabolism, and separate determination of the unbound fraction in the locust brain [111]. Conserved mechanisms between efflux transporters in insects and mammals are also supported by the transcriptomic identification of a human P-glycoprotein in locusts and the kinetic analysis of transport by a PgP substrate in the ex-vivo model [112]. Recently, machine-learning chemoinformatic models have been applied to predict the uptake of compounds into the ex-vivo locust brain [113]. Based on the published, and the availability of further, experimental data, the correlation of the ex-vivo locust with mammalian blood–brain barrier models appears satisfactory. In future research, the test compounds should first be applied to the ex-vivo locust brain model to obtain the kinetic parameters for uptake and potential metabolic breakdown. This approach might then be valuable for combining the subsequently generated DNT assay data with physiologically based kinetic modelling for risk assessment [108,109].

The migratory locust is not only a classical model organism for studies of nervous-system development and electrophysiological analysis of neural circuits [49]. It is becoming more tractable for genetic analysis. The complete genome is sequenced, which has enabled new approaches, such as targeted gene-editing by the CRISPR/Cas-9 system [114,115]. The application of this technique to mutate genes that code for guidance factors and their intracellular signal transduction molecules could resolve more specifically the effects of developmental neurotoxicants on these targets.

## 9. Outlook

Since the very beginnings of written records in antiquity, the swarming locust has been an iconic animal as a devastating agricultural pest [116]. Consequently, it appears somewhat ironic to introduce the locust embryo as a promising model for testing DNT, a procedure for the benefit of human health. Even though this approach has, so far, only been used to track the formation of a specific axonal pathway, it can be expanded to other endpoints (Table 3). Despite the impressive progress in the development of alternative DNT testing methods, the pioneer neuron assay seems to be the first assay that quantifies pathfinding errors of single, identifiable neurons in concentration-response curves. I would also like to emphasize the utility of other invertebrates for predictive DNT assessment. After evaluation of their successful performance using a panel of test substances, they should be considered for inclusion as complementary alternative models in DNT test batteries.

## Figures and Tables

**Table 1:** Comparison of chemical-induced effects on the human nervous system and pioneer axon assay of locust embryos.

Compound	For Humans	For Pioneer Axon Assay
methyl mercury	DNT-positive	DNT-positive
sodium(meta) arsenite	DNT-positive	DNT-positive
rotenone	DNT-positive	DNT-positive
valproic acid	DNT-positive	DNT-positive
chlorpyrifos	DNT-positive	DNT-negative!
Chlorpyrifos oxon	DNT-positive	DNT-negative!
paracetamol	DNT-negative	DNT-negative
penicillin G	DNT-negative	DNT-negative
saponin	DNT-negative	DNT-negative
DMSO	DNT-negative	DNT-negative
sodium dodecyl sulfate	DNT-negative	DNT-negative
hydrogen peroxide	DNT-negative	DNT-negative

**Table 2:** Comparison of test compound-induced effects on pioneer axon pathway and resazurin assay. Especially in cases where the upper limits of concentrations–response curves did not reach half maximal inhibition, the highest concentration was used as IC50 and served as the most sensitive endpoint (MSE) [11]. The ratio of the most sensitive endpoints (MSE ratio) served as criteria for the distinction between DNT-positive and DNT-negative compounds [11,74]. According to the prediction model [38], the MSE ratio of DNT-negative compounds is below the threshold of 4.1.

Compound	IC50 Axon Pathway	IC50 Viability/MSE Ratio
methyl mercury	4.9 µM	67.22 µM/13.72
sodium(meta) arsenite	9.3 µM	100 µM/10.75
rotenone	24.44 nM	>400 nM/16.37
valproic acid	5.18 mM	>100 mM/19.31
chlorpyrifos	>3.0 mM	>3.0 mM/1.0
Chlorpyrifos oxon	0.9 mM	2.5 mM/2.78
paracetamol	>10.0 mM	>10.0 mM/1.0
penicillin G	>10.0 mM	>10.0 mM/1.0
saponin	17.55 µM	13.86 µM/0.88
DMSO	729.9 mM	1354.0 mM/1.86
sodium dodecyl sulfate	73.26 µM	119.0 µM/1.62
hydrogen peroxide	4.0 mM	13.36 mM/3.34

**Table 3:** Potential for the development of adverse outcome pathways (AOPs) as assayed in locust embryos. To establish the AOP, disruption of pathway can be analyzed with small molecule ligands.

Endpoint	Initiating Event of AOPs	References
Axon outgrowth	Disruption of cGMP signaling	[38]
	Disruption of Fasciclin II binding *	[62]
Axon pathfinding	Blocking of semaphorin signaling	[64,65,66,67]
	Disruption of cGMP signaling	[38]
Neuron migration	Disruption of NO/cGMP signaling	[101,102,103]
	Disruption of HO/CO signaling	[102,103]
Synaptogenesis Excitability and EPSP recording	Blocking of AChE activity	[72]
	Blocking of channels and receptors	[106,107]
Programmed cell death	Blocking of caspases	[105]
Expression of neurogenic genes	Disruption of Notch signaling	[49,50,52,53,54]

* Fasciclin II has structural homology with N-CAM.
